# Bat aggregational response to pest caterpillar emergence

**DOI:** 10.1038/s41598-021-93104-z

**Published:** 2021-07-01

**Authors:** Ján Blažek, Adam Konečný, Tomáš Bartonička

**Affiliations:** Department of Botany and Zoology, Faculty of Science, Masaryk University, Kotlářská 2, 611 37 Brno, Czech Republic

**Keywords:** Ecology, Ecosystem services, Forest ecology, Zoology

## Abstract

Moths (Lepidoptera) are major agricultural and forest pests in many parts of the world, including Europe, with many causing great economic damage to crops, horticultural plants, stored items, and wool products. Here, we focus on two ecologically similar inchworms, *Operophtera brumata* and *Erannis defoliaria*, known for their high foliage consumption during the spring emergence of caterpillars. We hypothesise that bats could play a role in reducing pests such as caterpillars by switching to this abundant emerging prey. At two infested and one control forest sites, caterpillars were sampled during spring to determine levels of infestation. At the same time, bat flight activity was monitored during the peak in caterpillar abundance. During the spring caterpillar outbreak, we collected faecal samples of forest-dwelling bats capable of using gleaning. The majority of samples were positive for our focus species, being 51.85% for *O. brumata* and 29.63% for *E. defoliaria* faecal samples. The foraging activity of two gleaning bats, *Myotis nattereri* and *Myotis bechsteinii*, increased at both infested sites, but not at the control site, during caterpillar emergence, as did foraging of *Plecotus auritus/austriacus*, which used both gleaning and aerial hawking. We conclude that both specialists and occasional gleaners, which prefer different prey but are able to switch their foraging strategies, aggregate at sites during pest emergence and, as such, our results confirm the high potential of bats to reduce numbers of pest species such as caterpillars.

## Introduction

A predator’s effect on prey populations is generally studied using numerical responses^1,2^ driven by two mechanisms, migration of predators to sites with high prey concentrations (aggregational response) and predator reproduction, which results in a delayed increase in the density of predators^3^. While a numerical response is usually expected for predators that specialise on certain prey, an aggregational response can be observed even in opportunistic generalist predators such as insectivorous bats. Bats, therefore, have the potential to act as an important pest control species, even if they only forage on such species occasionally, e.g., during outbreaks. Indeed, ecosystem services provided by bats are now considered of high economic importance^4,5^.

Given the high diversity of bat species and their varying foraging strategies, they have the potential to consume a wide range of arthropod pests within crop fields or agroforests^6,7^. A number of recent studies have identified arthropod species consumed by insectivorous bats and defined their role as regular predators^8–11^, especially following the rapid development of DNA metabarcoding methods that allow for the processing of multiple samples to identify different bat prey taxa^12,13^. However, the consumption of a pest does not indicate a predator’s ability to control its numbers^7^.

To declare a bat species capable of biocontrol on a certain pest, one needs to identify (1) that the bat forages on a pest regularly, (2) that it aggregates at a site where the pest accumulates, possibly shifting to a more effective foraging strategy, and (3) that the bat consumes enough of the pest to prevent massive outbreaks. To date, biocontrol has only been confirmed for *Tadarida brasiliensis* in the USA^14,15^, and there have been no comprehensive studies on the ability of bats to exert biocontrol in Europe. Although some studies have shown that bats can forage on pests at the stage of the outbreak^5,16,17^, there is still a lack of studies showing that bats actively aggregate at sites of mass pest occurrence to forage intensively upon it.

Every year, European deciduous forests are threatened by population overgrowth of pest defoliators, particularly moths^18,19^. While bats are significant predators of moths^20^, consumption of adult moths does not lead to an automatic reduction in caterpillars^7^, which are the main cause of foliage damage^14^. Theoretically, bats are potentially important predators of pest caterpillars as they have a suitable foraging strategy and occupy the same habitat as the pest during its emergence. Aside from three studies, regular consumption of caterpillars by Central European bats has not been well documented. Andreas et al.^21^, for example, detected caterpillars in the diet of three forest-dwelling bat species (*Plecotus auritus*, *Myotis bechsteinii,* and *Myotis nattereri*) using morphological analysis of faeces, while Vesterinen et al.^22^ detected pest caterpillars of the geometrid *Agriopis aurantiaria* in the diet of *Myotis brandtii*. Hope et al.^23^ molecularly confirmed and morphologically identified the consumption of caterpillars by gleaning bat *M. nattereri*. Finally, while there have been several recent studies focused on the ecosystem services provided by bats^10,24–26^, none have focused on the consumption of caterpillars causing significant damage to forests.

To assess the biocontrol potential of gleaning bats regarding forest pest inchworms, we employed a holistic approach that involved (1) monitoring bat acoustic activity during pest emergence to confirm aggregation at overpopulation sites; (2) detecting pest caterpillars in bat droppings using morphological and molecular analysis; and (3) evaluation of caterpillar density and foliage damage in infested and control forest sites to estimate pest impact.

## Results

### Caterpillar availability

**Figure 1:**
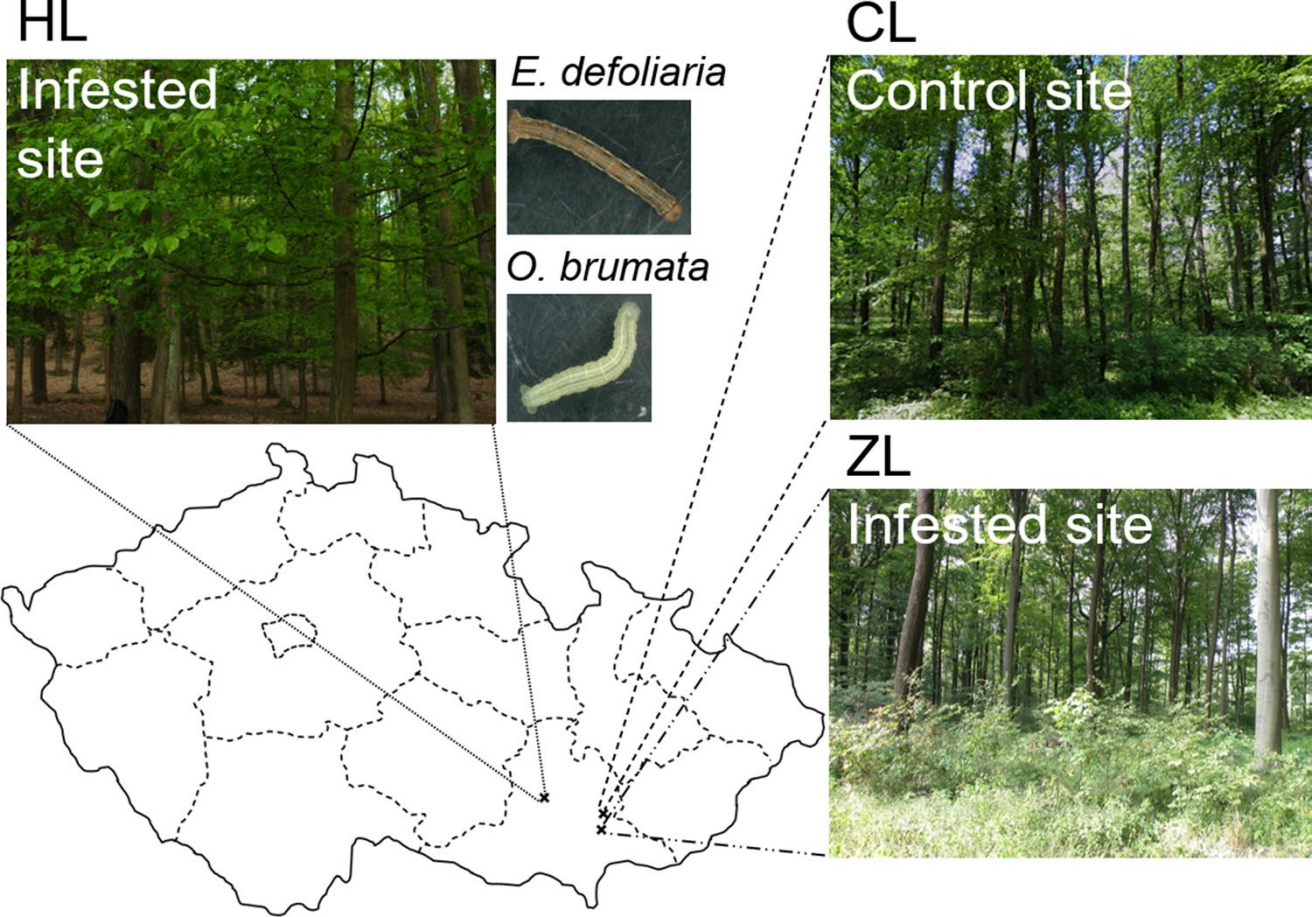
Sampling sites. ZL = Ždánický forest, HL = Holedná game reserve (both sites with regular massive caterpillar outbreaks), CL = control site. Map was generated in MS PowerPoint v2104 (www.microsoft.com).

**Figure 2:**
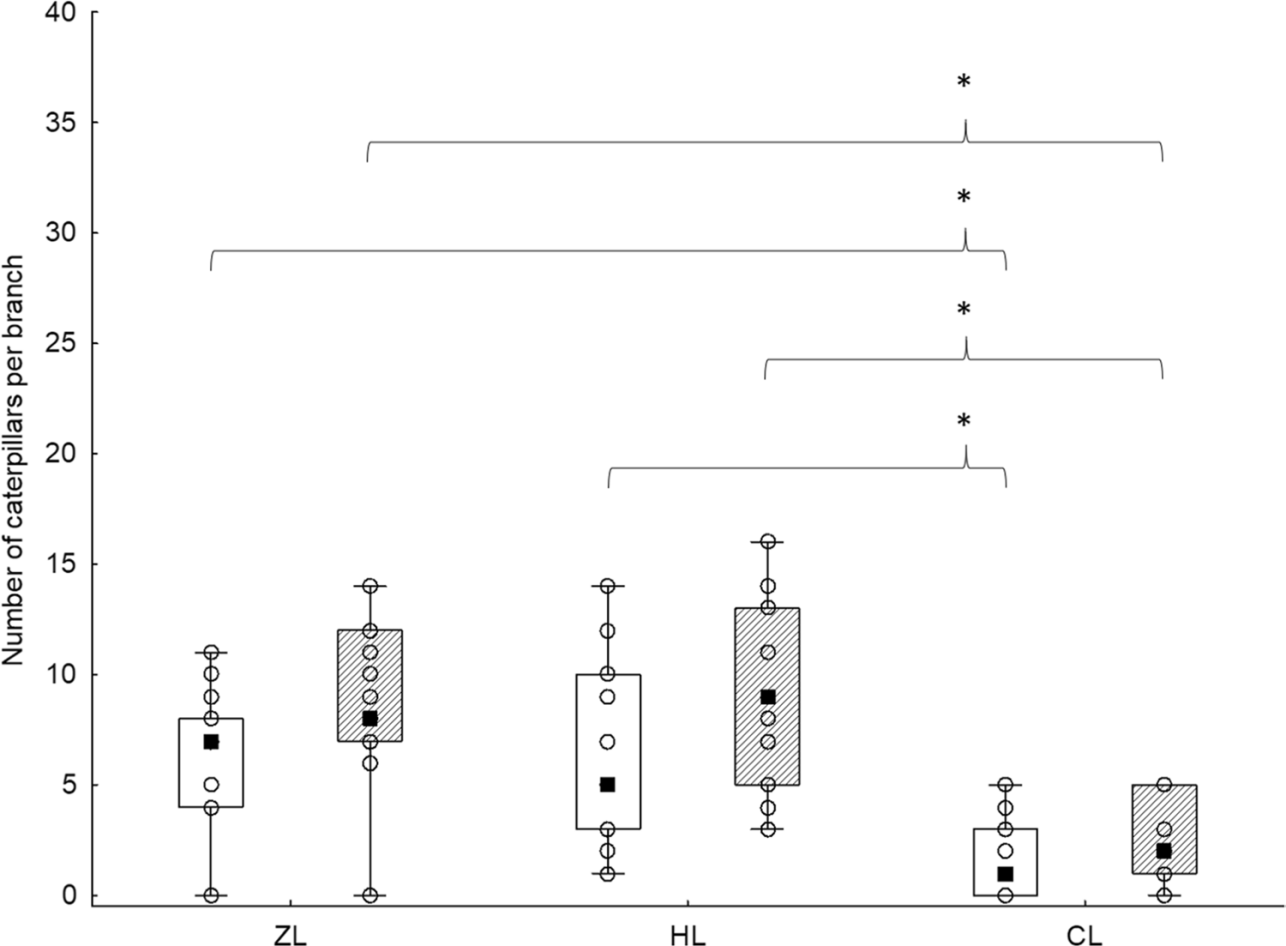
Number of caterpillars per branch (n = 15). Clear box = *E. defoliaria* and *O. brumata* caterpillars, Dashed box = all caterpillars; ■ = median; box = Q1–Q3; whiskers = range; ○ = source data. Data were compared using the Kruskal–Wallis H test (‘*’*p* ≤ 0.001).

The study was conducted on three sites (Fig. 1). Sites ZL and HL were marked by foresters as regularly infested by pest caterpillars. Site CL was picked as the control site because no such outbreaks occurred there. Two caterpillar species, *Erannis defoliaria* and *Operophtera brumata*, dominated at each site (ZL 68.06%, HL 59.17%, CL 52.5%), with *O. brumata* always more abundant than *E. defoliaria*. At ZL and HL, there were almost three times more *O. brumata* and ten times more *E. defoliaria* than at CL (Table 1). Despite a similar pest caterpillar proportion at all sites, the number of pest caterpillars per branch was significantly lower at CL than ZL or HL (median: 1, 7 and 5, respectively; H_(2,45)_ = 20.91, *p* < 0.001; Fig. 2). In comparison to lepidopteran caterpillars, other arthropods were the minority at all study sites (Table 1).

**Table 1:** Arthropod prey availability at the three study sites.

Site	Caterpillars	*E. defoliaria*	*O. brumata*	Other caterpillars	Other Arthropoda
CL	Sampled	3	18	16	3
	Average per branch	0.2	1.2	1.07	0.2
ZL	Sampled	37	61	31	15
	Average per branch	2.47	4.07	2.07	1
HL	Sampled	39	61	66	3
	Average per branch	2.6	4.07	4.4	0.2

*CL* control site, *ZL* Ždánický forest, *HL* Holedná game reserve.

### Foliage loss during pest emergence

**Figure 3:**
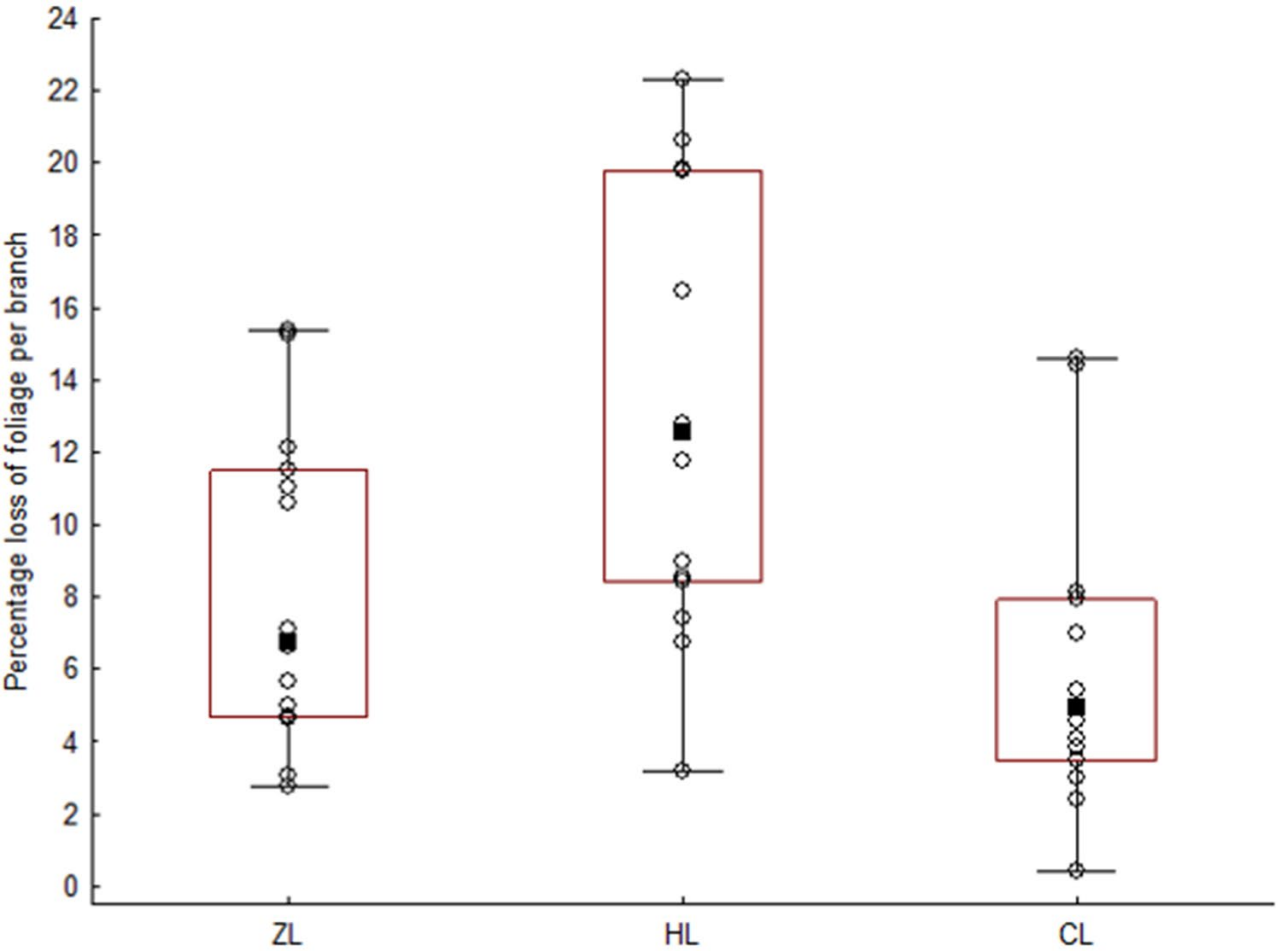
Foliage loss per branch at Ždánický forest (ZL), the Holedná game reserve (HL), and the control site (CL), n = 15 at each site. ■ = median; box = Q1 – Q3; whiskers = range; ○ = source data.

GLMs indicated that foliage loss was significantly influenced by both total number of *No. Caterpillars* (Wald χ^2^(1) = 9.05, *p* < 0.01) and *SITE* (Wald χ^2^(2) = 18.04, *p* < 0.001). Foliage loss was lowest at CL (Median: 4.9%; MAX: 14.6%; MIN: 0.4%; Fig. 3), which corresponded with the relatively low number of caterpillars detected (Table 1). Overpopulation sites with a higher number of caterpillars per branch displayed significantly higher foliage loss (ZL = Median: 6.8%; MAX: 15.4%; MIN: 2.8%; HL = Median: 12.5%; MAX: 22.3%; MIN: 3.2%; Fig. 3).

### Bat flying activity

**Figure 4:**
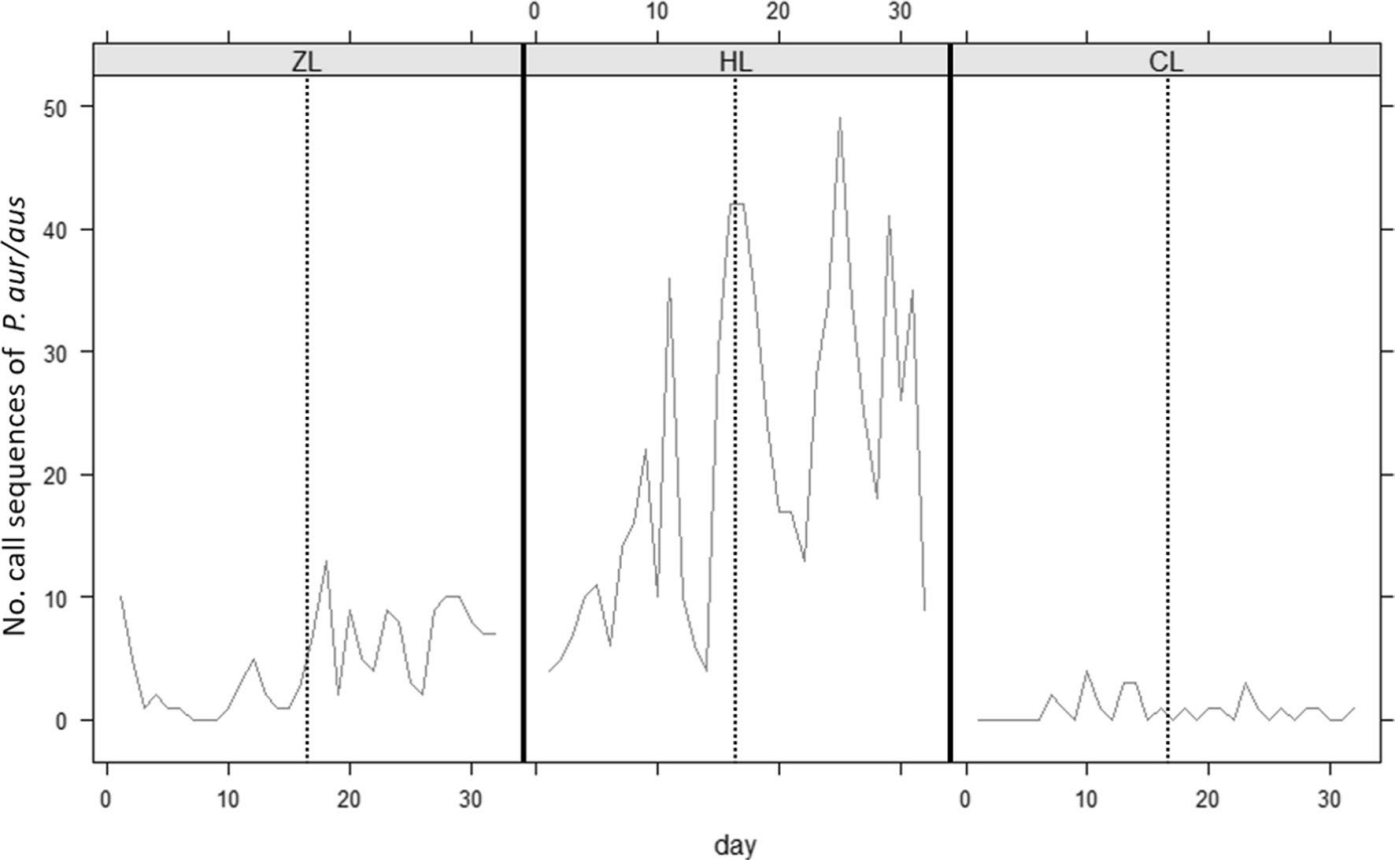
Flying activity of *P. auritus*/*austriacus* at the sample sites, presented as recorded call sequences per day. The dotted line divides the time periods “before” and “during” peak abundance.

**Figure 5:**
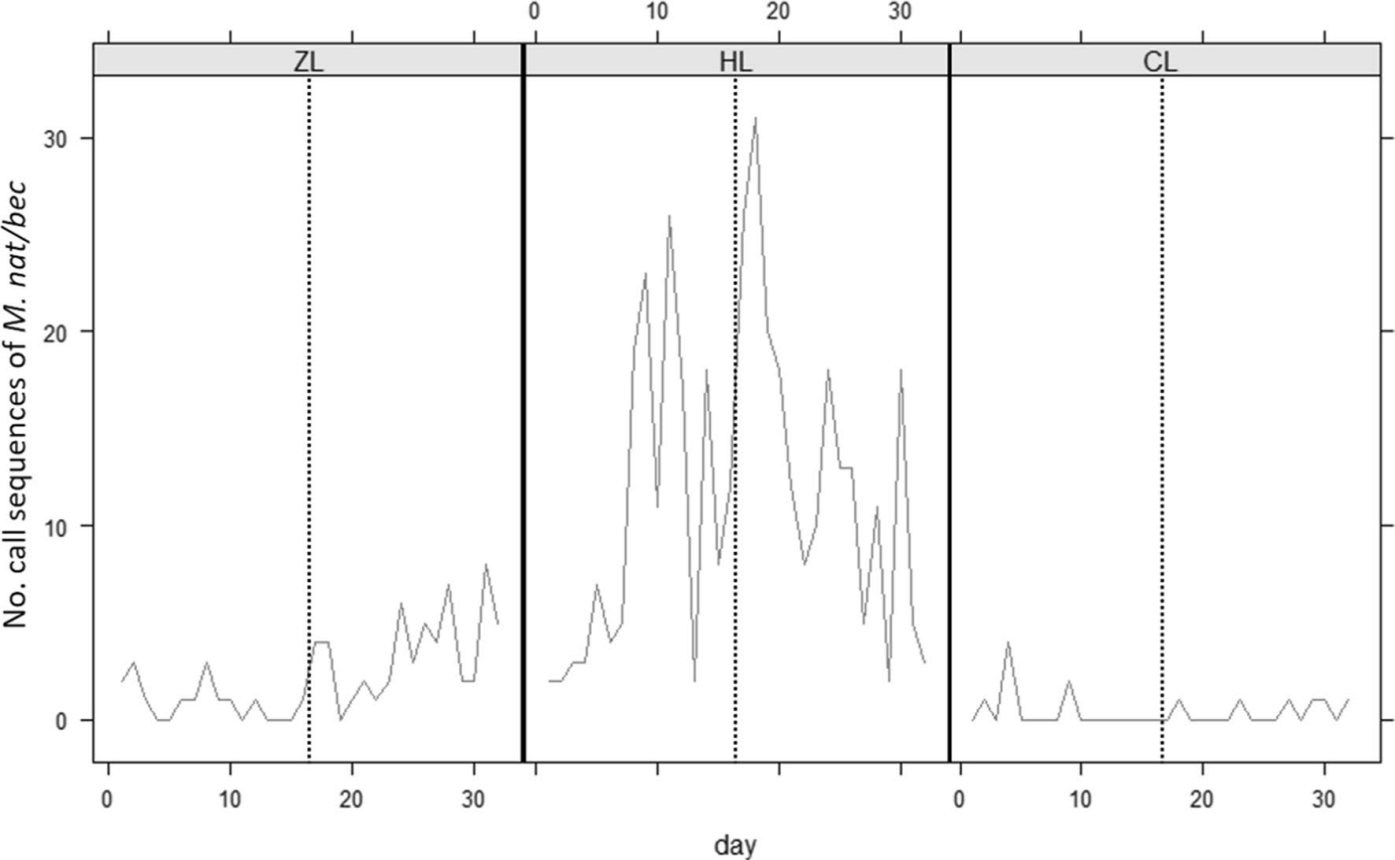
Flying activity of *M. nattereri*/*bechsteinii* at the sample sites, presented as recorded call sequences per day. The dotted line divides the time periods “before” and “during” peak abundance.

Analysis of bat activity was focused on *Plecotus auritus* and *Plecotus austriacus* (grouped together as *Plecotus*; opportunistic gleaners)*,* and *Myotis bechsteinii* and *Myotis nattereri* (grouped together as *Myotis*; solely gleaners) due to their known use of gleaning. Bat activity differed significantly between sites for both bat groups (*Plecotus*: Wald χ^2^(2) = 2.62*10^11^, *p* < 0.001; *Myotis*: χ^2^(2) = 4.21*10^11^, *p* < 0.001), with higher flying activity recorded at HL and ZL than CL (Figs. 4, 5). There was also a significant increase in flying activity for both bat groups in relation to caterpillar abundance before and during the abundance peak at HL and ZL (*Plecotus*: χ^2^(1) = 58, *p* < 0.001; *Myotis*: χ^2^(1) = 11, *p* < 0.001), with no increase observed at CL (Figs. 4, 5).

### Dietary analysis

Morphological analysis confirmed the remains of caterpillars in more than half of all faecal samples (51.85%; Table 2), with most being found in the droppings of *M. nattereri* (57.14% occurrence). As morphological analysis does not allow identification of species, we focused more on molecular confirmation for this purpose. Metabarcoding analysis confirmed 189 arthropod species of which 129 species were *Lepidoptera.* From the 129 *Lepidoptera* identified, we found 23 pest caterpillar species (termed “certain caterpillar”) and 26 pest species that had juvenile and adult stages that overlapped during the sampling period, meaning we could not say whether they were eaten as adult moths or caterpillars (termed “possible caterpillar”). All 81 samples were positive for at least one certain pest caterpillar, and the majority (74.07%) were positive for at least five certain pest caterpillar species.

**Table 2:** Percentage occurrence of morphologically identified caterpillar remains and molecularly confirmed pest caterpillars. As the life stages of several pest species overlapped over the study period, we could not say whether they were consumed as adult moths or as caterpillars; hence, these are marked as “p” (possible caterpillars). Pest species that occur only as caterpillars are marked as “c” (certain caterpillar). Species are sorted from the highest occurrence to the lowest.

		All samples (n = 81)	*P. aur/aus* (n = 33)	*M. nat* (n = 7)	*M. bec* (n = 41)
Morphological identification of caterpillars		51.9%	48.5%	57.1%	53.7%
Caterpillar	Molecular confirmation				
p	*Panolis flammea*	98.8%	97%	100%	100%
p	*Conistra erythrocephala*	96.3%	93.9%	100%	97. 6%
p	*Drymonia ruficornis*	93.8%	97%	85.7%	92.7%
p	*Polyploca ridens*	76.5%	84.9%	100%	65.9%
c	*Dichonia convergens*	72.8%	60.6%	85.7%	80.5%
p	*Selenia tetralunaria*	70.4%	57.6%	100%	75.6%
p	*Conistra rubiginosa*	61.7%	51.5%	85.7%	65.9%
p	*Tortrix viridana*	60.5%	51.5%	85.7%	63.4%
p	*Orthosia cerasi*	59.3%	57.6%	57.1%	61%
p	*Orthosia gothica*	56.8%	60.6%	57.1%	53.7%
c	*Operophtera brumata*	51.9%	54.6%	57.1%	48.8%
p	*Agriopis marginaria*	49.4%	45.5%	57.1%	51.2%
c	*Alsophila aceraria*	48.2%	30.3%	57.1%	61%
c	*Minucia lunaris*	48.2%	51.5%	28.6%	48.8%
p	*Hypomecis roboraria*	45.7%	36.4%	42.9%	53.7%
p	*Orthosia incerta*	45.7%	42.4%	85.7%	41.5%
c	*Agriopis aurantiaria*	45.7%	27.3%	85.7%	53.7%
p	*Diurnea fagella*	44.4%	54.6%	57.1%	34.2%
c	*Ennomos quercinarius*	43.2%	27.3%	85.7%	48.8%
c	*Griposia aprilina*	40.7%	33.3%	14.3%	51.2%
p	*Eupithecia abbreviata*	38.3%	48.5%	28.6%	31.7%
c	*Catocala sponsa*	38.3%	15.2%	42.9%	56.1%
p	*Lithophane ornitopus*	37%	33.3%	71.4%	34.2%
c	*Pandemis cerasana*	32.1%	21.2%	42.9%	39%
p	*Alsophila aescularia*	30.9%	30.3%	42.9%	29.3%
p	*Ectropis crepuscularia*	30.9%	21.2%	57.1%	34.2%
c	*Erannis defoliaria*	29.6%	33.3%	14.3%	29.3%
c	*Pennithera firmata*	29.6%	21.2%	42.9%	34.2%
p	*Lycia pomonaria*	27.2%	18.2%	71.4%	26.8%
c	*Amphipyra berbera*	27.2%	33.3%	14.3%	24.4%
p	*Lycia hirtaria*	25.9%	24.2%	42.9%	24.4%
p	*Phigalia pilosaria*	25.9%	21.2%	0%	34.2%
c	*Dioryctria abietella*	22.2%	18.2%	57.1%	19.5%
c	*Colotois pennaria*	21%	15.1%	42.9%	22%
c	*Dendrolimus pini*	21%	12.1%	57.1%	22%
p	*Cabera pusaria*	19.8%	24.2%	14.3%	17.1%
c	*Cosmia trapezina*	19.8%	12.1%	28.6%	24.4%
p	*Conistra rubiginea*	17.3%	12.1%	42.9%	17.1%
c	*Amphipyra pyramidea*	17.3%	3%	57.1%	22%
c	*Dryobotodes eremita*	17.3%	12.1%	14.3%	22%
c	*Archips xylosteana*	16.1%	9.1%	14.3%	22%
c	*Mesogona acetosellae*	13.6%	24.2%	0%	7.3%
c	*Eudemis profundana*	11.1%	9.1%	0%	14.6%
p	*Agriopis leucophaearia*	9.9%	3%	0%	17.1%
p	*Biston stratarius*	8.6%	6.7%	0%	12.2%
p	*Orthosia cruda*	8.6%	3%	14.3%	12.2%
c	*Ancylis mitterbacheriana*	8.6%	9.1%	0%	9.8%
c	*Archips oporana*	7.4%	6.1%	14.3%	7.3%
p	*Eupithecia lanceata*	4.9%	9.1%	0%	2.4%

The most frequent certain caterpillar found was that of *Dichonia convergens*, with caterpillars of our focal species *O. brumata* the second most frequent (51.85%; Table 2). Occurrence of *O. brumata* was similar across bat species, with 54.55% of *Plecotus* spp., 57.14% of *M. nattereri,* and 48.78% of *M. bechsteinii* faecal samples. In comparison, the frequency of occurrence of the second focus species, *E. defoliaria*, was just 29.63% in all samples. In bat species, *E. defoliaria* was detected in 29.27% of *M. bechsteinii*, 33.33% of *Plecotus* spp., and only 14.29% of *M. nattereri* faecal samples, its lower representation probably reflecting its lower availability in the environment. Other important pests of deciduous trees detected included *Agriopis aurantiaria* at 45.68% and *Agriopis leucophaearia* at 9.88% (Table 2).

Four species that may have been hunted by bats as both moth and caterpillar stages (*Panolis flammea*, *Conistra erythrocephala*, *Drymonia ruficornis*, *Polyploca ridens*) were molecularly confirmed more frequently than all other prey species that were definitely at the caterpillar stage at the time of the study (76–99%oc; Table 2).

## Discussion

Our results confirmed that *Myotis* species, such as *Myotis nattereri* and *Myotis bechsteinii*, aggregate at sites of mass caterpillar occurrence and systematically forage upon them. As far as we know, this is the first observation of an aggregational response by bats against caterpillars. On the other hand, the aggregation of a predator that specialises on gleaning is not so surprising if we are focusing on caterpillars. What may be more important in this case, therefore, is the confirmation of mass aggregation by a predator that displays multiple foraging strategies and can switch strategies during the year^27^, i.e., *Plecotus spp*. Since the different arthropod groups differ in availability during the year, we hypothesised that generalist facultative predators, such as bats, could play an important role in reducing pest invasion (biocontrol), with the importance of different species increasing with their ability to utilise food types in proportion to their availability^15^, as was indeed observed in this study.

Flying activity of these gleaning bats increased significantly with peak caterpillar abundance, while activity remained unchanged at the control site throughout the observation period. Until now, relatively few studies have reported the emergence of adult pest insects as an aggregational response trigger in bats^26,28,29^. Spatio-temporal matching between emergent moths and foraging bats, with bat activity increasing significantly with moth abundance, has previously been observed by Charbonnier at el.^30^, and an aggregational three-dimensional response was also confirmed by Krauel et al.^31^, who observed higher bat activity at higher altitudes when the abundance of migratory moths was high.

We observed slight differences in the diet of the two focus *Myotis* species in this study, with dietary patterns similar to those found by Gregor & Bauerova^32^, Swift^33^, and Taake^34^ for *M. nattereri*, and Andreas et al.^21^ and Vaughan^35^ for *M. bechsteinii*. Both showed similar foraging strategies, concentrating on stable prey resources in highly cluttered habitats by gleaning on leaf surfaces, using prey-generated sound alone for detection^36^, though both can employ aerial hawking in exceptional cases^37^. In comparison, *Plecotus auritus* is better able to utilise the nonregular distribution of ephemeral food resources thanks to its more common use of aerial hawking^38,39^. While only 10–40% of *P. auritus* diet is composed of terrestrial arthropods caught by gleaning^33,40^, it is generally accepted that the species uses aerial hawking in more than 50% of prey capture attempts^41^, making *P. auritus* a more generalist feeder than both *Myotis* species.

Caterpillars represent an excellent food source for gleaning bats, particularly during their peak abundance in spring; nevertheless, confirmation of their consumption by bats is almost absent in the literature. Our data show that over half of the bats sampled had morphologically identified caterpillar remains in their faeces. This is relatively high compared to the study of Andreas et al.^21^, who found 25.9%oc in *M. bechsteinii*, 18.8%oc in *M. nattereri,* and 12.1%oc in *P. auritus* faecal samples. However, Andreas et al.^21^ sampled from March to November to increase the amount of dietary data obtained, and these bats focus on caterpillar feeding during their summer emergence, which is consistent with opportunistic foraging on caterpillars. Furthermore, 100% of our samples were molecularly confirmed as containing remains of pest caterpillars, with *Operophtera brumata* confirmed at around 50%oc in all bat species studied, only *Dichonia convergens* occurring more frequently at 60–85%oc. Puig-Montserrat et al.^26^ noted that *Pipistrellus pygmaeus* predation had a significant impact on the rice borer moth *Chilo supressalis*, despite it only occurring in 50%oc of faecal samples. This would indicate that insectivorous bats preying on a vast array of arthropod species, are able to reduce pest species when their percentage occurrence in the diet is around 50%.

While *O. brumata* and *Erannis defoliaria* caterpillars were the most frequently occurring gleanable prey at our sites, they were not the most frequently taken pest taxa in the faecal samples, the most foraged taxa belonging to pest moths with overlapping stages. Overall, four “possible caterpillar” species were taken more often than any “certain caterpillar” pest species (Table 2). While consumption of adult stages does not automatically lead to pest reduction^7^, it may be still important for biocontrol if their occurrence in faecal samples is suitably high^26^. More effective suppression would occur in cases of predation by multiple species^25^. This is likely to be the case for *O. brumata*, which is targeted during both its spring and autumn emergences by multiple bat species^23,42^.

We measured foliage loss at all three sites as a proxy for tree damage and caterpillar activity/abundance. Foliage loss also acts as an indicator of a tree’s reaction to environmental stress and may serve as an indicator of continued vitality^43^. As expected, foliage loss was higher at infested sites, with levels of 6.8% at ZL and 12.5% at HL compared with 4.9% at the CL site. While such damage levels may seem insignificant in relation to the defoliation caused by other pests (e.g., the European gypsy moth *Lymantria dispar*^44^; the nun moth *Lymantria monachal*^45^), the reduction is visible in the first stages of foliage deficit and significantly slows down tree growth, with 1% foliage loss equivalent to a ~ 1% decrease in growth rate^43,46^. Furthermore, geometrid caterpillars tend to consume undamaged leaves and avoid previously damaged leaves^47^. Foliage damage in early spring, when trees have a higher specific leaf area and leaves are young, results in a greater reduction in leaf life span than if it occurred in species with a lower specific leaf area or with mature leaves^48^. Thus, we conclude that defoliators such as geometrids do not differ in their effects on leaf life span from other leaf miners or gall inducers. In species such as spruce, oak, and beech, levels of foliage deficit can increase even further in the years subsequent to droughts and heatwaves^49,50^. Indeed, thanks to climate change, droughts and heatwaves have increased noticeably in Europe over recent years, as has the occurrence of massive caterpillar infestations^51^, which has significantly increased the focus on developing adequate methods of prognosis^52^.

Our results showed that foliage loss corresponded with the number of all caterpillars at the sites. While the infested sites were shown to have significantly more *O.brumata* and *E. defoliaria* caterpillars than the CL site, the overall caterpillar assemblage at each infested site differed, with twice as many nontarget caterpillars found at HL. Populations of *O.brumata* and *E. defoliaria* are relatively easy to define spatially due to their weak dispersal ability, the females being flightless^53^, making dispersal between forest patches highly unlikely. We observed similarly high numbers of *O.brumata* and *E. defoliaria* at ZL and HL, but low numbers at CL. While both ZL and HL have stable populations of these pest geometrids, based on forester reports of repeated outbreaks, the CL population is either just starting to grow or is being suppressed by other predators (e.g. other arthropods or birds). While a higher caterpillar abundance and species diversity was likely to be the cause of the high level of foliage loss observed at HL (the highest of all sites studied), we were unable to definitively explain the high foliage loss, despite high bat flying activity, because we did not monitor the foraging activity of other predators, such as birds and arthropods, which could only have been done using exclosure experiments (see Bohm et al.^54^).

In conclusion, our results confirmed that pest caterpillars play an important role in the diet of both solely gleaning bat species, such as *M. nattereri* and *M. bechsteinii*, and that of species switching between foraging strategies, such as *Plecotus* spp. Furthermore, we were able to show that the aggregation and flying activity of such species increased significantly at infested sites during the spring emergence of caterpillars. Foliage damage was higher at infested sites compared with the control, with differences in the degree of damage directly related to the overall number of all caterpillar species present, rather than the abundance of our focus species, *O. brumata* and *E. defoliaria*. Nevertheless, one can assume that damage levels would have been higher still in the absence of bat predation. As both direct and indirect leaf damage is likely to increase in the face of climate change, we suggest that further experimental studies are needed as regards the relevance of bats in pest control, with special emphasis on their role in controlling pest caterpillar species.

## Methods

### Importance of model pest species

Oaks (*Quercus* spp.) are the most widespread and the most caterpillar-damaged deciduous tree species in Czech forestry^55^. In relation to biodiversity, oaks are considered a key species as they provide key habitat structures for cavity nesters and many other species^55^. Two of the most significant Central European oak pests are the geometrid moths *Opheroptera brumata* and *Erannis defoliaria*. While these do not completely destroy forests, they cause a significant loss of leaf area and have been the cause of several defoliation events over the past decades, thereby increasing the trees susceptibility to other types of damage, withering and reduced wood quality^56^. As drought conditions have increased over recent years with climate change, the negative impacts of tree pests on oaks have also increased^52,57^. Both geometrids have a similar annual life cycle, with caterpillars emerging in spring (April–May) and adult moths emerging in autumn (October–November)^58^. Multi-year cycles occur every 7–11 years, when massive overpopulation and severe defoliation may occur^52^. Such outbreaks tend to be the result of (1) coincidental caterpillar emergence with sprouting of oaks due to higher early spring temperatures^59^ and, especially in more recent years, (2) increased time for mating during warmer winters.

### Caterpillar availability

Three agricultural deciduous forests situated in southern Moravia, Czech Republic, were chosen for this study, two where spring outbreaks of geometrid caterpillars have repeatedly been observed in the past (Ždánický forest [ZL]; 350 m a. s. l.; 49.02°N, 17.02°E; the Holedná game reserve [HL]; 325 m a. s. l.; 49.21°N, 16.53°E) and one pest-free control site (CL; 350 m a. s. l.; 49.09°N, 17.02°E) (Fig. 1). At all three sites, the forest is predominantly made up of *Quercus robur*, *Q. petraea,* and *Carpinus betulus*, with less frequent occurrence of *Fagus sylvatica*.

The presence and size of caterpillars was checked on ten random trees at HL every week over April 2017. At the beginning of May, caterpillars were present on all control trees, suggesting that peak caterpillar abundance was imminent. Two separate sampling periods were established (group variable CATERPILLAR STATUS), the “before the peak” period lasting from 15 to 30 April, when caterpillars were present in low numbers and of small size (< 5 mm) and probably not yet attractive prey, and “during peak” period from 1–16 May, when peak abundance occurred. In the “during the peak” period, we sampled all available sedentary prey (caterpillars and other arthropods) from five randomly selected trees (three branches of similar size on each tree) at each site using the beating method^60^. All caterpillars were determined according to the key of Macek et al.^58^ and the website UKMOTHS^61^.

### Foliage loss

Foliage loss was evaluated in mid-June after most of the caterpillars had pupated. Foliage damage was assessed on five randomly selected deciduous trees at each site, with ten leaves from three branches sampled from each tree (150 leaves per site). All leaves were scanned and the leaf coverage calculated (in cm^2^) using the software package ImageJ v1.5 (https://imagej.nih.gov/; NIH, USA). In the case of damaged leaves, we estimated the original coverage and the subsequent percentage loss in foliage coverage (in cm^2^). Samplings were carried out in accordance with Czech law No 289/1995.

### Bat flying activity

At each site between mid-April and mid-May 2017, an SM4BAT detector (Wildlife Acoustics, Inc., Maynard, USA) equipped with an SMX-II microphone was set to record from sunset to sunrise. The detectors were attached to a tree trunk 6 m above the surrounding vegetation so that the branches did not block the microphones. The power supply and SD cards were replaced every 10 days. For the trigger system, the amplifier gain was set to 36 dB to ensure that only those events that were most likely to be bats were recorded. Recordings obtained in real-time were analysed with the Sonochiro v4 (http://sonochiro.biotope.fr/) software package (Biotope, Méze, France), using the northern temperate library of recordings (classifier). Software settings, post-processing and data analysis followed that of Bartonička et al.^62^. We focused on bat species that have the potential to forage on sedentary caterpillars, either solely gleaning predators (*Myotis bechsteinii, Myotis nattereri*) or both gleaning and aerial hawking predators (*Plecotus auritus, Plecotus austriacus*)^21^. For further analysis, we used the number of call sequences of two groups of foliage gleaners, i.e., the pair *P. auritus*/*austriacus* and *M. bechsteinii/nattereri*. Sequences were also grouped based on period (CATERPILLAR STATUS; before, during), and sampling site (SITE; ZL, HL, CL). Each echolocation sequence was determined on the species (or species pair) level with a minimum confidence index (MCI) ranging from 0 to 10 (cf.^62^). For each species, 15% of its sequences (range 3–485) were manually identified (Supplementary Table S1). The only exception was *P. pipistrellus* and *P. pygmaeus*, for which only 4% of the recordings were manually identified due to the high number of recordings. The MCI was determined at a value where 90% overlap was found in the determination of species identification using SonoChiro and manual identification^62,63^. To arbitrarily set the lowest value of the minimum confidence index, Batsound 4.12 (Pettersson Elektronik AB, Uppsala, Sweden) and Barataud^64^ were used. All studied species, i.e., *P. auritus*, *P. austriacus*, *M. nattereri,* and *M. bechsteinii*, were repeatedly mist-netted at all three sites.

### Dietary analysis

Bats were mist-netted at the overpopulation sites between April and May 2017 only. In total, we sampled 41 M*. bechsteinii*, seven *M. nattereri*, 32 *P. auritus,* and one *P. austriacus*. Each bat was placed separately in a linen bag and left for four hours to defecate, whereupon it was released at the same locality^21^ and the faeces stored separately in 96% ethanol. Linen bags were washed at high temperatures between uses. The bats were captured and handled under a license from the South Moravian Regional Authority, Permit no. JMK 63761/2017. Tomáš Bartonička is authorized to handle free-living bats under Certificate of Competency No. CZ01297 (§17, Czech law No 246/1992). Each dropping sample then underwent morphological analysis, with each pellet placed on a petri dish with 96% ethanol, disrupted using a dissection needle and tweezers and inspected under a microscope (magnification ranging from 1.6× to 5.6×) to confirm the remains of caterpillars. The percentage occurrence of identified taxa (%oc) was estimated following McAney et al.^65^. For every twenty samples, we added one negative control sample (four in total) to test possible contamination. All instruments were sterilized by fire and the workstation including microscope was sterilized and bleached between the samples.

After morphological analysis, all 85 samples (including negative controls) underwent DNA isolation using the NucleoSpin DNA Stool kit (MACHEREY–NAGEL GmbH & Co. KG, Düren, Germany), following the manufacturer’s “Protocol for fresh or frozen stool samples” version April 2016/Rev. 01. There were four DNA isolation sessions (3 × 24 samples and once 13 samples) during each session, one negative control was included into the process.

Subsequent PCR amplification of the 157 bp section of the COI mitochondrial gene was performed using the primers ZBJ‐ArtF1c: 5′‐GATATTGGAACWTTATATTTTATTTTTGG-3′ and ZBJ‐ArtR2c: 5′‐WACTAATCAATTWCCAAATCCTCC‐3′^66^. These primers are frequently used in studies focusing on bat diet barcoding and are known to be biased towards Lepidopteran and Dipteran taxa^67^, and hence beneficial for our study. Unambiguous identification of subsequently pooled samples was ensured through unique combinations of MID-tagged primers. PCR thermal cycling conditions were as follows: 15 min at 95 °C, followed by 40 cycles of 30 s at 94 °C, 90 s at 50.5 °C and 90 s at 72 °C, followed by a final incubation of 10 min at 72 °C. The PCR products were visualised on a 1% agarose gel and purified using standard EXOCIP purification. Negative control samples showed no signs of amplification and thus were not sequenced. The purified PCR products were then pooled appropriately to achieve an equal DNA concentration (about 11 ng/ul) and sent to a commercial sequencing facility. Sequencing was processed using the MiSeq Reagent Kit version 3 system (Illumina) by the SEQME company (Dobříš, Czech Republic; www.seqme.eu).

### Bioinformatics analysis

We merged raw paired-end 250 bp reads (ca. 3 M reads) and made a quality check with the USEARCH v10 (https://www.drive5.com/) software package, using the algorithms -fastq_mergepairs, -fastq_trunctail 30 and -fastq_minovlen 50. This resulted in approximately 1.5 M merged reads (2.85% were filtered during the process). We then removed the tagged primers using the Python program Cutadapt v1.15 (https://cutadapt.readthedocs.io/), which also demultiplexed samples according to the tags^68^, thereby rejecting 51.1% of the merged reads. When using Cutadapt, we discarded all reads shorter than 150 bp and longer than 165 bp, thereby rejecting 1% of demultiplexed reads. We then dereplicated reads using the USEARCH fastx_uniques algorithm, using the option minuniquesize 2. The final read count was > 700,000, with an average of 8 737 reads per sample (MAX: 53,898; MIN: 1323). We then applied the USEARCH UNOISE3 algorithm to cluster these unique reads into zOTUs and automatically filter chimaeras. The reads were then mapped back to the original samples using the USEARCH otutab algorithm.

To match zOTUs with appropriate taxa at the highest level possible, we compared our data with the GENBANK database, using a minimum sequence similarity of 98%^69^. When several prey species provided an equal best match, selection was based on the species known to be present in the study area. Moth species that feed on agricultural trees such as oak, hornbeam, spruce, or pine were determined as pest species.

### Statistical analysis

To calculate the significance of sampling site and caterpillar status on bat flying activity, we used the following model for *Plecotus* (*P. auritus/austriacus*) and *Myotis* (*M. bechsteinii/naterreri*): log(sequences) = α + *SITE* + *CATERPILLAR STATUS* + *SITE: CATERPILLAR STATUS*. The Wald Chi-Squared test was then applied to reveal significant differences between sites for both bat groups. Analysis of flying activity was carried out using the lattice and geepack packages in R v. 4.0.3^70^, using a poisson distribution model with log link. We used the geeglm model function with an AR1 correlation structure to counter autocorrelation in the time series data. Shapiro–Wilk normality tests were undertaken using the STATISTICA 12 statistics package (StatSoft, Inc, Tulsa, USA), as were Kruskal–Wallis H tests (caterpillar comparison among sites) and generalised linear model (GLM) for the analysis of foliage loss, based on percentage foliage loss per branch (%cm^2^) with *SITE* (CL, HL, ZL) and total *No. caterpillars* as explanatory variables. The total number of caterpillars includes all caterpillars sampled on foliage and not just the focal species.

## Supplementary Information

Supplementary Information.

## Data Availability

The datasets generated and analysed during the current study are available from the corresponding author on reasonable request.
