# Supplementary material for: Bat aggregational response to pest caterpillar emergence
Source: Sci Rep. 2021 Jul 1;11:13634. doi: 10.1038/s41598-021-93104-z (PMC8249614; doi:10.1038/s41598-021-93104-z)
Supplement: Supplementary file 1 — Supplementary Information. [file 41598_2021_93104_MOESM1_ESM.docx]

**Supplementary Table S1**

Bat sequences recorded in the three studied sites (CL = Control site, ZL = Ždánický forest, HL = Holedná game reserve). Minimum confidence index (MCI) where the probability of correct species determination was higher than 90%. Probability (Prob, in %) of correct determination on manually processed sequences. NbseqMan — number of manually processed sequences/the percentage of all sequences per species at the minimum confidence index. When species identification was not possible, bat sequences were attributed to the genus or the group of species proposed by the software and are not included.

|  |  |  |  |  |  |  |
| --- | --- | --- | --- | --- | --- | --- |
| **Species/Species pairs** | **MCI** | **Prob** | **NbseqMan** | **CL** | **ZL** | **HL** |
| *Barbastella barbastellus* | 4 | 92 | 485/15 | 727 | 2496 | 16 |
| *Eptesicus nilssonii* | 6 | 91 | 12/15 | 16 | 64 | 0 |
| *Eptesicus serotinus* | 4 | 93 | 18/16.2 | 20 | 50 | 41 |
| *Myotis bechsteinii* | 8 | 90 | 150/31.4 | 13 | 412 | 53 |
| *Myotis daubentonii* | 8 | 91 | 51/15.1 | 2 | 332 | 3 |
| *Myotis emarginatus/alcathoe* | 3 | 90 | 120/15.2 | 23 | 678 | 87 |
| *Myotis myotis/blythii* | 3 | 91 | 110/15.1 | 118 | 598 | 11 |
| *Myotis mystacinus/brandtii* | 6 | 90 | 149/15 | 15 | 925 | 54 |
| *Myotis nattereri* | 8 | 90 | 130/78.8 | 8 | 127 | 30 |
| *Nyctalus leisleri* | 4 | 91 | 7/18.9 | 3 | 32 | 2 |
| *Nyctalus noctula* | 0 | 97 | 291/15 | 74 | 1475 | 397 |
| *Pipistrellus nathusii/kuhlii* | 2 | 92 | 22/15 | 79 | 51 | 12 |
| *Pipistrellus pipistrellus* | 0 | 93 | 395/4 | 2750 | 6720 | 435 |
| *Pipistrellus pygmaeus* | 0 | 90 | 495/4 | 560 | 10418 | 1343 |
| *Plecotus auritus/austriacus* | 4 | 90 | 348/23.8 | 53 | 1283 | 128 |
| *Vespertilio murinus* | 4 | 90 | 3/33.3 | 2 | 7 | 0 |
|  |  |  |  |  |  |  |
